# Decreased plating efficiency, proliferation and osteogenic differentiation of synovial fluid mesenchymal progenitors as a marker of severity of juvenile idiopathic arthritis

**DOI:** 10.1186/ar3636

**Published:** 2012-02-09

**Authors:** Elvira Lazic Mosler, Marija Jelusic-Drazic, Danka Grcevic, Ana Marusic, Natasa Kovacic

**Affiliations:** 1 Laboratory for Molecular Immunology, University of Zagreb School of Medicine, Zagreb HR-10000, Croatia; 2 Department of Pediatrics, Division of Pediatric Rheumatology and Immunology, University Hospital Centre Zagreb, Zagreb HR-10000, Croatia; 3 Department of Physiology and Immunology, University of Zagreb School of Medicine, Zagreb HR-10000, Croatia; 4 Department of Research in Biomedicine and Health, University of Split School of Medicine, Split HR-21000, Croatia; 5 Department of Anatomy, University of Zagreb School of Medicine, Zagreb HR-10000, Croatia

## Background

Juvenile idiopathic arthritis (JIA) is a rheumatic pediatric disease characterized by synovial inflammation in one or more joints [1]. Inflammation results in hyperplastic changes of the synovium, destruction of articular cartilage and subchondral osteoresorption. Murine models of arthritis revealed impaired osteogenic/chondrogenic differentiation of synovial mesenchymal progenitors via inflammation-induced activation of NF-κB [2].

We aimed to explore frequency, plating efficiency and osteoblastogenic potential of synovial mesenchymal progenitors and correlate them with intensity of local and systemic inflammation in patients with JIA.

## Materials and methods

Synovial fluid cells were collected from 19 patients with oligoarticular JIA (oJIA) and 8 patients with poliarticular JIA (pJIA), plated in density 1.5 × 10^6^/mL in 24-well plates, and cultured in αMEM + 10% FCS. Osteoblastogenesis was stimulated by the addition of 50 μg/ml ascorbic acid and 5 mmol β-glycerophosphate. To exclude inflammatory and hematopoietic cells, adherent cells were passaged three times, and osteoblastogenesis again induced in fourth passage (P4). Osteoblastogenesis was assessed by intensity of alkaline phospatase (AP) histochemical staining. In addition, osteoblast (Runx2, AP, OPG, RANKL) and cytokine/chemokine (IL-1, IL-4, CCL2, CCL4 and MIP1α) gene expression were assessed in P4 osteoblastogenic cultures.

## Results

Plating efficiency of synovial mesenchymal progenitors was decreased in patients with pJIA in comparison to patients with oJIA. Passage was successful only in 3 (37.5%) pJIA patients, and 18 (94.7%) oJIA patients. Plated at equal density, P4 synovial adherent cells from pJIA patients formed less fibroblastic colonies. Osteoblastogenesis was higher in children with oJIA than in children with pJIA, both from primary synovial cells (median 1119.08; IQR 476.57-1470.26 vs. 141.58; IQR 14.47-237.50, arbitrary units, p < 0.005, Mann-Whitney test), and P4 cells (median 1162.00; IQR 102.00 to 5484.50 vs. 12.00; IQR 6.00-307.37, arbitrary units, p < 0.05). Osteoblastogenesis from primary synoviocytes negatively correlated with erythrocyte sedimentation rate (ρ= -0.4139, p = 0.03), and synovial concentration of IL-17 (ρ= -0.4174, p = 0.04). Expression of osteoprotegerin and CCL2 was decreased in P4 osteoblastogenic cultures from pJIA in comparison with oJIA patients (p < 0.05).

## Conclusions

Severe forms of JIA are characterized by decreased proliferation, osteogenic differentiation and immunoregulatory potential of synovial mesenchymal cells, correlating with inflammatory activity.
